# Dengue Virus Serotype 3, Karachi, Pakistan

**DOI:** 10.3201/eid1301.060376

**Published:** 2007-01

**Authors:** Bushra Jamil, Rumina Hasan, Afia Zafar, Kevin Bewley, John Chamberlain, Valerie Mioulet, Moira Rowlands, Roger Hewson

**Affiliations:** Aga Khan University Hospital, Karachi, Pakistan; † Health Protection Agency, Salisbury, United Kingdom

**Keywords:** Dengue serotype 3, outbreak, Karachi, letter

**To the Editor:** The global prevalence of dengue fever (DF) has grown dramatically in recent decades; DF is now endemic to >100 countries (*1*). Dengue hemorrhagic fever (DHF), a potentially lethal complication of dengue virus infection, was first recognized in Asia in the 1950s and is now a leading cause of hospitalization and death among children (*1*). During the past decade, DHF epidemics have occurred in China, Sri Lanka, India, the Maldives, Bangladesh, and Pakistan (*2*–*4*).

In Pakistan, an outbreak of DHF was first reported in Karachi in 1994 (*4*). Currently, 15–20 patients with DF or DHF are admitted each year to the Aga Khan University Hospital (AKUH), a tertiary care referral center in Karachi. Many more cases, however, may go unrecognized. Ours is the first report of dengue virus serotype 3 in Pakistan.

From September through December 2005, at least 3 major hospitals in Karachi, including AKUH, had a sudden increase in the number of patients with signs consistent with the World Health Organization definition of DHF: high fever, rash, epistaxis, gum bleeding, liver dysfunction, and thrombocytopenia (platelets <100,000/mm^3^); most had evidence of capillary leakage in the form of raised hematocrit and pleural effusion with or without ascites (*5*). Because in Pakistan, Crimean-Congo hemorrhagic fever (CCHF) is an important differential diagnosis for hemorrhagic fever, most patients seen at AKUH received care in strict isolation and were empirically treated with ribavirin. At time of admission, blood samples were collected for serologic testing for dengue virus and reverse transcription (RT)–PCR testing for CCHF virus. The first 5 samples, collected during the initial 2 weeks of the outbreak, were also sent to the Special Pathogens Reference Unit, Centre for Emergency Preparedness and Response, Health Protection Agency, Salisbury, United Kingdom, for diagnostic confirmation. In the absence of a local surveillance and disease notification system, the number of patients with suspected DHF at different hospitals in Karachi could not be ascertained.

Of the 106 patients who had a clinical diagnosis compatible with DHF (*5*), 9 (8.5%) died and 97 (91.5%) recovered. Patients with possible DF (fever, mild thrombocytopenia with platelets >100,000/mm^3^) were not admitted and were treated as outpatients. Dengue virus infection was confirmed for 42 of the 106 patients. Serum samples from 39 patients contained anti–dengue virus immunoglobulin M (IgM) antibody (Chemicon, Temecula, CA, USA). Diagnosis for 6 of these patients was confirmed by using immunoblot tests (Dengue IgM Blot and Dengue IgG Blot, Genelabs Diagnostics, Singapore). Of the 9 patients who died, 6 had dengue IgM and IgG according to immunoblot testing, and 3 had dengue IgM according to ELISA. Diagnoses for 3 additional patients were confirmed by RT-PCR.

An RT-PCR assay specific for dengue viruses (*6*) was used to amplify the C/PrM/M region of the genome and produced PCR products of the expected size in 3 patient samples: 2 (K1 and 2) from Karachi and 1 (B) from Balochistan. The PCR products were sequenced, and data were subsequently placed in GenBank under accession numbers DQ469827 for D3418-05 (patient K1), DQ469828 for D3419-05 (patient K2), and DQ469826 for D3417-05 (patient B). These data were compared with those in databases by using the basic local alignment search tool for nucleotides (blastn), with default settings (*7*). For each sequence analyzed, the lowest Expect (E) value showed significant similarity with a dengue serotype 3 isolate from India in 2004 (DQ323042). A phylogenetic tree was constructed with a collection of dengue sequences (Appendix Table). Phylogenetic relationships between sequences are depicted in the Figure. Sequences from the 2005 outbreak are most similar to those from Indian strains of dengue serotype 3, which were isolated in Delhi.

**Figure:**
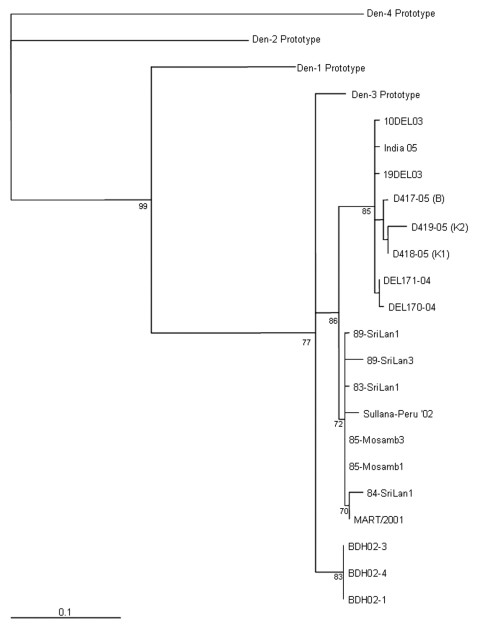
Maximum-likelihood phylogenetic tree of established dengue virus 3 serotypes and new sequences from Pakistan identified in this study. The tree is based on a 238-nt sequence alignment comprising the C/PrM/M gene (nucleotides: 179–417 dengue virus 3 prototype [NC_001475]). The bar shows the number of substitutions per bases weighted by the Tamura-Nei algorithm. Horizontal distances are equivalent to the distances between isolates; numbers at nodes indicate support values for the branch of the tree inferred at the node. The origins of the sequences used to reconstruct this tree are detailed in the Table.

An unexpected finding was the detection, at both AKUH and the UK Special Pathogens Reference Unit, of dengue-3 and CCHF virus RNA in the sample from patient B. CCHF is endemic to the rural Balochistan province of Pakistan, where DF has been documented (*8*). In the absence of information on the current dengue situation in Balochistan and given the increasing dengue activity in Karachi, a similar increase can be assumed for Balochistan. Possible introduction of dengue serotype 3 in a CCHF-endemic area resulted in dual infection in patient B, who essentially had clinical and laboratory features compatible with DHF. Patient B received ribavirin and recovered. Our results suggest that the 2005 outbreak of DHF in Karachi, Pakistan, was caused by strains of dengue virus serotype 3 related to those circulating in India (*9*).

## Supplementary Material

Appendix Table Dengue stains and sequences used in phylogenetic analysis
