# Supplementary material for: Dengue Virus Serotype 3, Karachi, Pakistan
Source: Emerg Infect Dis. 2007 Jan;13(1):182–3. doi: 10.3201/eid1301.060376 (PMC2725812; doi:10.3201/eid1301.060376)
Supplement: Appendix Table — Dengue stains and sequences used in phylogenetic analysis [file 06-0376_appT-s1.pdf]

Appendix Table. Dengue stains and sequences used in phylogenetic analysis

Dengue strain	Location	Year isolated	Accession no.
Dengue 1 prototype	NA*	NA	NC_001477
Dengue 2 prototype	NA	NA	NC_001474
Dengue 3 prototype	NA	NA	NC_001475
Dengue 4 prototype	NA	NA	NC_002460
83-SriLan1	Sri Lanka	1983	AF547225
84-SriLan1	Sri Lanka	1984	AF547229
85-Mosamb1	Mozambique	1985	AF547236
85-Mosamb3	Mozambique	1985	AF547238
89-SriLan1	Sri Lanka	1989	AF547230
89-SriLan3	Sri Lanka	1989	AF547227
MART/2001	Martinique	2001	AY099342
Sullana-Peru 6682-01	Peru	2002	AY079175
BDH02-1	Bangladesh	2002	AY496871
BDH02-3	Bangladesh	2002	AY496873
BDH02-4	Bangladesh	2002	AY496874
10DEL03	Delhi, India	2003	AY706097
19DEL03	Delhi, India	2003	AY706099
DEL170-04	Delhi, India	2004	DQ323041
DEL171-04	Delhi, India	2004	DQ323042
D3417-05 (B)	Balochistan, Pakistan	2005	DQ469826
D3418-05 (K1)	Karachi, Pakistani	2005	DQ469827
D3419-05 (K2)	Karachi, Pakistan	2005	DQ469828
India 2005	India	2005	DQ317393

*NA, not applicable for prototypes.
